# The safety of early discharge following transfemoral transcatheter aortic valve replacement under general anesthesia

**DOI:** 10.3389/fcvm.2022.1022018

**Published:** 2022-10-21

**Authors:** Ofir Koren, Vivek Patel, Siamak Kohan, Robert Naami, Edmund Naami, Zev Allison, Sharon Shalom Natanzon, Alon Shechter, Takashi Nagasaka, Ahmed Al Badri, Arvind Reddy Devanabanda, Mamoo Nakamura, Wen Cheng, Hasan Jilaihawi, Raj R. Makkar

**Affiliations:** ^1^Cedars-Sinai Medical Center, Smidt Heart Institute, Los Angeles, CA, United States; ^2^Bruce Rappaport Faculty of Medicine, Technion Israel Institute of Technology, Haifa, Israel; ^3^Internal Medicine, Kaiser Permanente Los Angeles Medical Center, Los Angeles, CA, United States; ^4^Internal Medicine, University Hospitals Cleveland Medical Center, Case Western Reserve University School of Medicine, Cleveland, OH, United States; ^5^School of Medicine, University of Illinois Chicago, Chicago, IL, United States; ^6^Sackler School of Medicine, Tel Aviv University, Tel Aviv, Israel; ^7^Department of Cardiology, Gunma University Hospital, Gunma, Japan; ^8^Heart Valve Center, NYU Langone Health, New York City, NY, United States

**Keywords:** transcatheter aortic valve replacement, general anesthesia, conscious sedation, length of stay, early discharge, safety discharge, mortality, predictors

## Abstract

**Background:**

There is growing evidence of the safety of same-day discharge for low-risk conscious sedated TAVR patients. However, the evidence supporting the safety of early discharge following GA-TAVR with routine transesophageal echocardiography (TEE) is limited.

**Aims:**

To assess the safety of early discharge following transcatheter aortic valve replacement (TAVR) using General Anesthesia (GA-TAVR) and identify predictors for patient selection.

**Materials and methods:**

We used data from 2,447 TEE-guided GA-TAVR patients performed at Cedars-Sinai between 2016 and 2021. Patients were categorized into three groups based on the discharge time from admission: 24 h, 24–48 h, and >48 h. Predictors for 30-day outcomes (cumulative adverse events and death) were validated on a matched cohort of 24 h vs. >24 h using the bootstrap model.

**Results:**

The >48 h group had significantly worse baseline cardiovascular profile, higher surgical risk, low functional status, and higher procedural complications than the 24 h and the 24–48 h groups. The rate of 30-day outcomes was significantly lower in the 24 h than the >48 h but did not differ from the 24–48 h (11.3 vs. 15.5 vs. 11.7%, *p* = 0.003 and *p* = 0.71, respectively). Independent poor prognostic factors of 30-day outcomes had a high STS risk of ≥8 (OR 1.90, 95% CI 1.30–2.77, *E*-value = 3.2, *P* < 0.001), low left ventricle ejection fraction of <30% (OR 6.0, 95% CI 3.96–9.10, *E*-value = 11.5, *P* < 0.001), and life-threatening procedural complications (OR 2.65, 95% CI 1.20–5.89, *E*-value = 4.7, *P* = 0.04). Our formulated predictors showed a good discrimination ability for patient selection (AUC: 0.78, 95% CI 0.75–0.81).

**Conclusion:**

Discharge within 24 h following GA-TAVR using TEE is safe for selected patients using our proposed validated predictors.

## Introduction

Transcatheter aortic valve replacement (TAVR) has become the most common procedure in the United States for symptomatic aortic stenosis (AS) patients across all risk profiles (1–3). Technological advancements and utilization of pre-procedural imaging for planning continues to improve TAVR outcomes. The operator’s familiarity aids in minimizing procedural complications and improves patient care (4, 5).

Despite the maturity of TAVR, only minimal changes in the length of hospitalization (LOS) have been noted over the years, with a wide variation among national and international centers (6–8). Unplanned 30-day hospital readmissions were directly associated with longer LOS and adversely impacted patient outcomes and health care costs (9, 10). However, these studies were not randomized or used propensity matched comparisons and may be subject to bias.

Numerous clinical and multimodal pathways were designed over the years to identify true low-risk patients for earlier and safe discharge, addressing known late complications such as bleeding, conduction disturbance, and acute kidney injury (7, 11, 12). In recent times, studies have shown that same-day discharge following TAVR, with conscious sedation, can be safe in select patient populations (13).

We hypothesized that discharging transfemoral-TAVR (TF-TAVR) patients performed under general anesthesia (GA-TAVR) using transesophageal echocardiography within 24 h of admission is safe and feasible and doesn’t pose a higher risk for major adverse events.

The study aims to assess the safety of early discharge following GA-TAVR and proposes risk-assessment predictors that would optimize patient selection for GA-TAVR.

## Materials and methods

### Study cohort and patients’ selection

We conducted a single-center retrospective study of 2,736 consecutive patients, with severe symptomatic AS, who underwent TAVR at Cedars-Sinai Medical Center between January 2016 and December 2021. A consensus decision of the multidisciplinary cardiac team determined the indication of TAVR for all patients and the interventional team and attending physicians determined the time of discharge.

**FIGURE 1:**
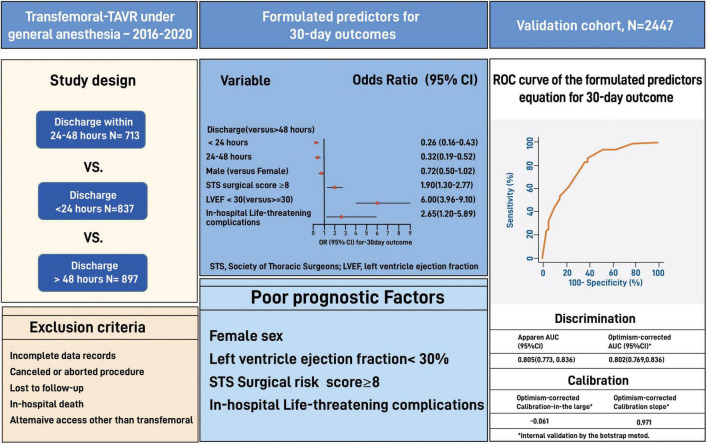
Central illustration.

Exclusion criteria included death during hospitalization, canceled or aborted procedure, utilization of non-transfemoral access sites, incomplete medical records, and if lost to follow up. Patients discharged to a rehabilitation center, skilled nurse facilities, and hospice were not excluded from the study to ensure real-life data. We used the data of 2,447 eligible patients to formulate a predictors equation and validated it on the entire cohort (Figure 1).

The study was approved by the Cedars-Sinai medical center institutional review board (IRB), which also waived the requirement to obtain informed consent due to the study’s retrospective nature.

### Definitions

Acute kidney injury, bleeding, vascular complications, and procedure-related complications were defined according to the updated Valve Academic Research Consortium-3 consensus document (VARC-3) (14). We defined procedure-related life-threatening complications to include any of the following adverse events: aortic valve annulus rupture, aortic dissection, cardiac arrest, cardiac tamponade, coronary obstruction, valve migration, conversion to open- heart surgery, major bleeding, and major vascular complications. We followed the valve academic research consortium-3 for the clinical outcome to include composite events of all-cause non-cardiac readmission, heart failure readmission, valve re-intervention, THV thrombosis, myocardial infarction, and unplanned percutaneous coronary intervention (PCI), cerebrovascular accident (CVA)/transient ischemic attack (TIA), new-onset atrioventricular block and atrial arrhythmias, pacemaker implantation, major bleeding, and vascular complications, acute renal failure, infective endocarditis, and death (14).

### Data collection

Demographic, procedural, and follow-up data were entered retrospectively by a dedicated team and extracted using the Cedars-Sinai electronic records systems (CS-link).

### Statistical analysis

The data is presented as a number of patients and percentage (%) for categorical variables and a median (IQR, interquartile range) for continuous variables. Patient characteristics were compared among discharge times (<24 h, 24–48 h and >48 h) using a Kruskal-Wallis test, chi-square test or Fisher’s exact test as appropriate. Pairwise comparisons were further carried out to compare discharge <24 h with 24–48 h and >48 h, and family-wise error rate was adjusted for inflation due to multiple comparisons using the Bonferroni correction method. Outcomes were reported as those occurring during the index hospitalization (“in-hospital”) and 30 days post discharge (“30-day”) as a means to prevent data analysis bias and emphasize each component of the outcome.

Univariate and multivariable analyses of discharge times (<24 h vs. 24–48 h, and < 24 h vs. >48) and 30-day outcomes were performed using a logistic regression model. Covariates considered in multivariable analyses were chosen *a priori* including age, sex, body mass index (BMI), Society of Thoracic Surgeons (STS) risk score, Kansas City Cardiomyopathy Questionnaire (KCCQ), left ventricle ejection fraction (LVEF), severe mitral and tricuspid regurgitation, urgent procedure, bicuspid aortic valve, use of intra-aortic balloon pump (IABP), in-hospital complications, procedure-related life-threatening complications, the use of contrast volume, and hours of stay in the intensive cardiac care unit.

Variable selection was performed using a stepwise variable selection procedure based on Akaike Information Criterion (AIC) (15). The model with the minimum AIC was chosen for analysis in order to minimize the loss of information. A forward stepwise analysis was used and a probability entry threshold value of *p* < 0.001 was set for all variables. In multivariable analyses, multicollinearity was assessed by the variance inflation factor. The performance of the formulated predictors of 30-day outcomes was assessed with measures of discrimination and calibration (16). Discrimination was assessed with a receiver operating characteristic (ROC) curve along with the area under the ROC curve (AUC, c-statistic). The calibration of the formulated predictors was evaluated with calibration-in-the-large, and the calibration slope proposed by Cox (17). Internal validation was performed by estimating and correcting possible overfitting and optimism in the predictors performance estimates (e.g., optimism-corrected c-statistic) using the bootstrap method with 1,000 replicates (18–20).

To address confounding caused by differing baseline patient characteristics, we adopted the inverse propensity treatment weighting (IPTW) using propensity score–based matching (PSM) and a greedy matching strategy with full matching in a one-to-one ratio including statistically significant variables from the logistic regression model and non-significant variables that might be related to unrecorded selections factors adopted from a parsimonious model (21, 22). The covariates included in the IPTW were age, sex, STS score, KCCQ performance and LVEF.

All statistical analyses were performed using SAS 9.4 (SAS Institute, Inc., Cary, North Carolina) and R package version 4.0.5 (23) with two-sided tests at a significance level of 0.05.

## Results

### Study population

We used the data of 2,736 TAVR patients at Cedars-Sinai medical center from 2016 to December 2021. Patients were eligible for the study if they were 18 years of age and older, had TAVR using general anesthesia, and used a new generation of commercial balloon-expanding and self-expandable valves.

We excluded 32 patients due to incomplete records, 4 patients due to procedure cancelation or abortion, and 24 patients who were lost to follow-up at their 30-day postoperative visit. We also excluded 35 (1.2%) patients who died during hospitalization and 194 patients who underwent non-transfemoral TAVR. TAVR was guided by transesophageal echocardiography (TEE) under general anesthesia in all patients.

**FIGURE 2:**
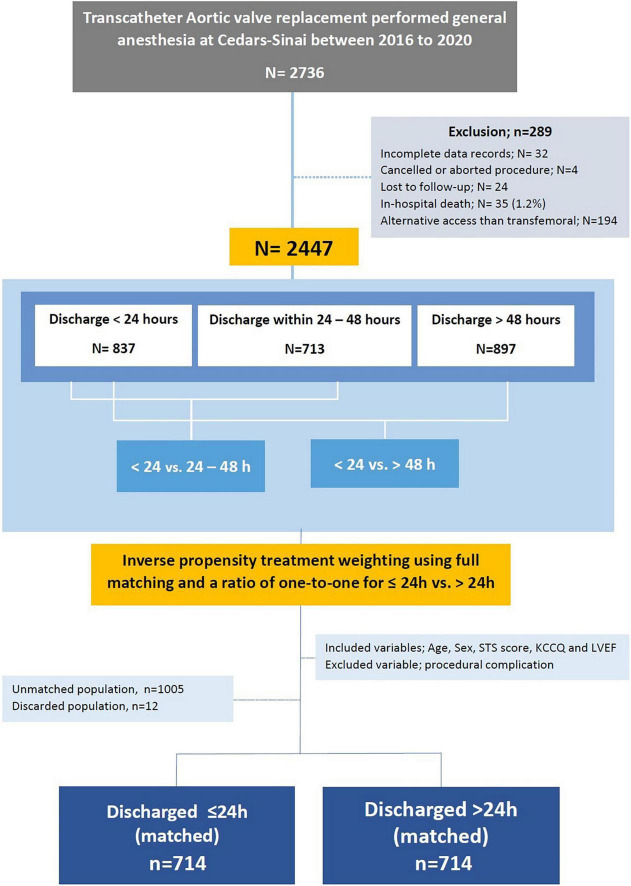
Study design.

Among 2,447 eligible patients, 837 patients were discharged within 24 h of admission (“24 h” group), 713 patients were discharged within 24–48 h (“24–48 h” group), and 897 patients were discharged after 48 h (“>48 h” group). Data from eligible patients was used to formulate predictors for patients selection and further validated using the bootstrap technique (Figures 1, 2).

### Patients baseline characteristics

#### 24 h group vs. >48 h group

Complete baseline characteristics are shown in Table 1. The 24 h group was significantly younger (79 vs. 82 years, *p* < 0.001), compromised of fewer females (34 vs. 40%, *p* = 0.005), more commonly demonstrated bicuspid aortic valve morphology (15.8 vs. 5.6%, *p* < 0.001), and had a lower rate of comorbidities including hypertension, hyperlipidemia, diabetes mellitus, peripheral artery disease (PAD), anemia, chronic kidney disease, chronic lung disease, coronary artery disease, CVA, and TIA (significant *p*-value for all).

**TABLE 1:** Patient characteristics stratified by discharge time.

	All patients (*N* = 2,447)	<24 h (A) (*N* = 837)	24–48 h (B) (*N* = 713)	>48 h (C) (*N* = 897)	*p*-value		
					Overall	A vs. B*	A vs. C*
Age (years), median (IQR)	81 (73–87)	79 (72–85)	82 (76–87)	82 (74–88)	<0.001	<0.001	<0.001
Female Sex	928 (37.9)	284 (33.9)	281 (39.4)	363 (40.5)	0.01	0.04	0.005
ADL index score, median (IQR)^β^	3.0 (2)	4.0 (2)	3.0 (3)	2.0 (2)	<0.001	<0.001	<0.001
KCCQ score, median (IQR)	54 (34–73)	58 (40–77)	55 (36–75)	47 (29–66)	<0.001	0.04	<0.001
5-min walk test (min), median (IQR)	7 (6–8)	6 (6–8)	7 (6–8)	7 (6–9)	<0.001	<0.001	<0.001
Albumin level (prior) g/dL, median (IQR)	4 (3.7–4.3)	4.1 (3.8–4.3)	4.1 (3.9–4.3)	3.8 (3.4–4.1)	<0.001	0.01	<0.001
BMI, median (IQR)	26 (23–30)	27 (24–31)	26 (24–30)	26 (23–30)	<0.001	1.00	<0.001
Hypertension	2,070 (84.6)	656 (78.4)	644 (90.3)	770 (85.8)	<0.001	<0.001	<0.001
Hyperlipidemia	1,285 (52.5)	370 (44.2)	468 (65.6)	447 (49.8)	<0.001	<0.001	0.04
Diabetes Mellitus	545 (22.3)	149 (17.8)	162 (22.7)	234 (26.1)	<0.001	0.03	<0.001
Anemia	143 (5.8)	23 (2.7)	21 (2.9)	99 (11.0)	<0.001	1.000	<0.001
Peripheral artery disease	261 (10.7)	76 (9.1)	92 (12.9)	93 (10.4)	0.04	0.03	0.61
CKD stage III or higher ^φ^	312 (12.7)	61 (7.3)	93 (13.0)	158 (17.6)	<0.001	<0.001	<0.001
Current dialysis	109 (4.4)	13 (1.5)	25 (3.5)	71 (7.9)	<0.001	0.03	<0.001
Chronic lung disease	321 (13.1)	91 (10.9)	96 (13.5)	134 (14.9)	0.03	0.22	0.02
Coronary artery disease	1011 (41.3)	272 (32.5)	312 (43.8)	427 (47.6)	<0.001	<0.001	<0.001
Myocardial infarction (prior)	263 (10.7)	67 (8.0)	64 (9.0)	132 (14.7)	<0.001	0.96	<0.001
CABG (prior)	331 (13.5)	77 (9.2)	107 (15.0)	147 (16.4)	<0.001	0.001	<0.001
CVA/TIA (prior)	160 (6.5)	41 (4.9)	50 (7.0)	69 (7.7)	0.08	0.20	0.06
Atrial fibrillation	570 (23.3)	143 (17.1)	189 (26.5)	238 (26.5)	<0.001	0.001	<0.001
RBBB or LBBB	307 (12.5)	72 (8.6)	100 (14.0)	135 (15.0)	<0.001	0.001	<0.001
Pacemaker implantation (prior)	271 (11.1)	87 (10.4)	80 (11.2)	104 (11.6)	0.61	1.00	0.66
HF Exacerbation in last two weeks	1,845 (75.4)	563 (67.3)	540 (75.7)	742 (82.7)	<0.001	<0.001	<0.001
NYHA class					<0.001	<0.001	<0.001
1	21 (0.9)	14 (1.7)	6 (0.9)	1 (0.1)			
2	309 (12.6)	161 (19.2)	71 (10.0)	77 (8.6)			
3	1,491 (60.9)	474 (56.6)	502 (70.4)	515 (57.4)			
4	626 (25.6)	188 (22.5)	134 (18.8)	304 (33.9)			
Balloon-expandable THV	1,985 (81.1)	748 (89.4)	560 (78.5)	677 (75.5)	<0.001	<0.001	<0.001
Use of large THV ^ψ^	585 (23.9)	200 (23.9)	176 (24.)	209 (23.3)	0.81	1.00	1.00
Bicuspid aortic valve	214 (8.7)	132 (15.8)	32 (4.5)	50 (5.6)	<0.001	<0.001	<0.001
Concomitant severe MR	109 (4.4)	17 (2.0)	16 (2.2)	76 (8.5)	<0.001	1.00	<0.001
Concomitant severe TR	122 (5.0)	23 (2.7)	21 (2.9)	78 (8.7)	<0.001	1.00	<0.001
Hgb level (prior), g/dL, median (IQR)	12.3 (10.9–13.6)	12.9 (11.5–1)	12.7 (11.3–13.8)	11.5 (10.0–12.8)	<0.001	0.13	<0.001
STS score, median (IQR)	4 (2–6)	3 (2–5)	4 (2–6)	5 (3–10)	<0.001	<0.001	<0.001
LVEF (%), median (IQR)	60 (51–65)	62 (56–66)	60 (55–66)	57 (40–64)	<0.001	0.08	<0.001
AV area (mm^2^), median (IQR)	0.7 (0.6–0.8)	0.7 (0.6–0.9)	0.7 (0.6–0.9)	0.7 (0.6–0.8)	0.001	1.00	0.002
AV peak gradient, median (IQR)	70 (63–81)	70 (64–80)	71 (64–81)	71 (61–82)	0.781	1.000	1.000
Coronary calcium score, median (IQR)	531 (289–1,424)	503 (271–1059.5)	525.5 (287–1,496)	568 (307–1,710)	<0.001	0.062	<0.001
AV calcium score, median (IQR)	1,725 (660–2,892)	1,597 (553–2,664)	1,847 (782–3,180)	1,721 (702–2,990)	0.01	0.005	0.04
Urgent TAVR	339 (13.8)	97 (11.6)	99 (13.9)	143 (15.9)	0.03	0.35	0.02
Use of IABP device during the procedure	17 (0.7)	11 (1.31)	3 (0.42)	3 (0.33)	0.03	0.13	0.05
Percutaneous suture closure device^Ω^	2,379 (97.2)	811 (96.9)	697 (97.7)	871 (97.1)	0.46	0.67	0.74
Fluoroscopy time (min), median (IQR)	14.6 (11–20)	15 (11.5–19.7)	13.1 (9.4–18.7)	15.4 (11.5–22)	<0.001	<0.001	0.08
Procedure duration (min), median (IQR)	46 (37–61)	45 (36–58)	45 (37–58)	48 (37–66)	<0.001	1.000	<0.001
Contrast volume (ml), median (IQR)	70 (50–87)	66 (49–82)	67 (50–85)	70 (50–100)	<0.001	0.39	<0.001
ICU length-of-stay (hours), median (IQR)	4 (0–7)	2 (0–4)	3 (0–4)	7 (5–8)	0.69	0.87	1.000
ECG abnormalities after TAVR	189 (7.7)	31 (3.7)	60 (8.4)	98 (10.9)	<0.001	<0.001	<0.001
Discharge location					<0.001	NA	<0.001
Home	2,358 (96.4)	837 (100)	713 (100)	808 (90.1)			
Hospice	3 (0.1)	0 (0)	0 (0)	3 (0.3)			
Rehabilitation	49 (2)	0 (0)	0 (0)	49 (5.5)			
Skilled nurse facility	37 (1.5)	0 (0)	0 (0)	37 (4.1)			

ADL, activity of daily life; BMI, body mass index; CKD, chronic kidney disease; CABG, coronary artery bypass graft; CVA/TIA, cerebrovascular accident/Transient ischemic attack; RBBB, right bundle branch block; LBBB, left bundle branch block; HF, heart failure; NYHA, New-York heart association; THV, transcatheter heart valve; MR, mitral regurgitation; TR, tricuspid regurgitation; Hgb, Hemoglobin; STS, society of thoracic surgeon; KCCQ, Kansas city cardiomyopathy questionnaire; LVEF, left ventricular ejection fraction; AV, aortic valve; TAVR, transcatheter aortic valve replacement; IABP, intra-aortic balloon pump; Ca, calcium score; ICU, intensive care unit. ^β^Katz Index of Independence in Activities of Daily Living (ADL). ^φ^Large THV referred to 29 mm Sapien valve or 34 mm Evolute valve. ^ψ^Based on GFR level according to the National Kidney Foundation (NKF). Data are presented as number of patients (column%) or median (IQR, interquartile range). *P*-value is calculated by Kruskal-Wallis test for continuous variables, and chi-square or Fisher’s exact test for categorical variables as appropriate. *Bonferroni adjusted p-values. ^Ω^Perclose ProGlide systems Abbott Vascular, Santa Clara, CA, USA.

The >48 h group comprised of more patients with high surgical risk (STS ≥ 8), low KCCQ (KCCQ < 50), low mean LVEF (57 vs. 62%, *p* < 0.001), and severe mitral and tricuspid regurgitation (*p* < 0.001 for both). Furthermore, the >48 h group more often required the use of an intra-aortic balloon pump (IABP) (1.3 vs. 0.3%, *p* = 0.04), exhibited longer procedure and fluoroscopy times, and used more contrast volume (*p* < 0.001, *p* = 0.08, *p* < 0.001, respectively).

The 24–48 h group shares baseline characteristics of both groups and includes older patients, including more females, higher rates of comorbidities, higher surgical risk, and lower KCCQ score than the 24 h group, however less pronounced when compared to >48 h group (Table 1).

#### Procedure-related outcome

In-hospital complications and procedure-related life-threatening complications occurred more in the >48 h group (15.0 vs. 7.0%, and 4.2 vs. 1.4%, *p* < 0.001 and *p* = 0.001) than in the 24 h group contributing mostly to a higher rate of unplanned percutaneous coronary intervention (PCI) (1.4 vs. 0%, *p* = 0.001), CVA or TIA (3.0 vs. 0.1%, *p* = 0.001), complete atrioventricular heart block (CAVB) (3.3 vs. 0.2%, *p* < 0.001), pacemaker implantation (7.3 vs. 4.9%, *p* = 0.04) and major vascular complications (2.0 vs. 0.1%, *p* < 0.001) (Table 2).

**TABLE 2:** Procedure related outcome stratified by discharge time.

	All patients (*N* = 2,447)	<24 h (A) (*N* = 837)	24–48 h (B) (*N* = 713)	>48 h (C) (*N* = 897)	*p*-value		
					Overall	A vs. B*	A vs. C*
**In hospital complications**							
Procedure-related complications	259 (10.6)	59 (7.0)	65 (9.2)	135 (15.0)	<0.001	0.58	<0.001
Procedure-related life-threatening complications ^ψ^	57 (2.3)	12 (1.4)	7 (1.0)	38 (4.2)	0.001	0.84	0.001
AV rupture or Aortic dissection	3 (0.1)	0 (0)	3 (0.4)	0 (0)	0.025	0.20	NA
Valve migration	6 (0.2)	1 (0.1)	1 (0.1)	4 (0.4)	0.391	1.00	0.75
Conversion to open heart surgery	5 (0.2)	0 (0)	3 (0.4)	0 (0)	0.025	0.20	NA
Coronary occlusion	9 (0.4)	1 (0.1)	1 (0.1)	4 (0.4)	0.109	0.93	0.25
Unplanned PCI	20 (0.8)	1 (0.1)	7 (1.0)	13 (1.4)	0.003	0.01	0.001
CVA or TIA	33 (1.3)	1 (0.1)	2 (0.3)	30 (3.0)	<0.001	1.00	0.001
Complete heart block	40 (1.6)	2 (0.3)	8 (1.1)	30 (3.3)	<0.001	0.10	<0.001
Pacemaker or ICD implantation	147 (6.0)	41 (4.9)	40 (5.6)	66 (7.3)	0.052	0.08	0.04
Major bleeding	15 (0.6)	7 (0.8)	2 (0.3)	6 (0.7)	0.100	0.43	0.127
Minor vascular complications ^€^	54 (2.2)	12 (1.4)	10 (1.4)	32 (3.6)	<0.001	0.27	<0.001
Major vascular complications ^€^	22 (0.9)	1 (0.1)	3 (0.4)	18 (2.0)	<0.001	0.68	<0.001
Aortic regurgitation ≥ 2 ^Ω^	5 (0.2)	1 (0.1)	1 (0.1)	3 (0.3)	0.635	1.00	1.00
Paravalvular leakage ≥ 2 ^Ω^	7 (0.3)	0 (0)	2 (0.3)	5 (0.6)	0.100	0.43	0.13
**30-day outcome**							
All cause re-admission^β^	34 (1.4)	7 (0.8)	7 (1.0)	20 (2.2)	0.03	1.00	0.04
Heart failure readmission	9 (0.4)	4 (0.5)	1 (0.1)	4 (0.4)	0.491	0.760	1.000
Valve re-intervention	4 (0.2)	3 (0.4)	0 (0)	1 (0.1)	0.21	0.51	0.71
Device thrombosis	15 (0.6)	3 (0.4)	7 (1.0)	5 (0.6)	0.23	0.41	1.00
Myocardial infarction or unplanned PCI	6 (0.2)	0 (0)	2 (0.3)	4 (0.4)	0.14	0.43	0.25
CVA or TIA	7 (0.3)	3 (0.4)	1 (0.1)	3 (0.3)	0.72	1.00	1.00
Heart block or new onset atrial arrhythmia	8 (0.3)	2 (0.2)	3 (0.4)	3 (0.3)	0.91	1.000	1.000
Pacemaker implantation	209 (8.5)	66 (7.9)	59 (8.3)	84 (9.4)	0.60	0.65	0.48
Major bleeding ^€^	6 (0.2)	0 (0)	1 (0.1)	5 (0.6)	0.04	0.93	0.12
Acute renal failure	15 (0.6)	5 (0.6)	1 (0.1)	9 (1)	0.09	0.45	0.73
Major vascular complications ^€^	3 (0.1)	1 (0.1)	1 (0.1)	1 (0.1)	0.99	1.00	1.00
Infective endocarditis	2 (0.1)	1 (0.1)	1 (0.1)	0 (0)	0.53	1.00	0.95
Cumulative events	318 (13.0)	95 (11.3)	84 (11.7)	139 (15.5)	0.04	0.71	0.03
Death	21 (0.9)	1 (0.1)	1 (0.1)	19 (2.1)	<0.001	1.00	<0.001
Outcome (cumulative event or death)	457 (18.7)	133 (15.9)	118 (16.5)	207 (23.1)	<0.001	0.98	<0.001

AV, aortic valve; PCI, percutaneous aortic valve implantation; CVA/TIA, cerebrovascular accident/Transient ischemic attack; ICD, implanted cardioverter defibrillator.

^ψ^Procedure-related life-threatening complications: AV annulus rupture, aortic dissection, cardiac arrest, cardiac tamponade, coronary obstruction, valve migration, conversion to open heart surgery, major bleeding, major vascular complications.

^Ω^Of at least moderate grade severity.

^€^Defined by the Valve Academic Research Consortium-2 consensus document (VARC-3).

Data are presented as number of patients (column%) or median (IQR, interquartile range). *P*-value is calculated by Kruskal-Wallis test for continuous variables, and chi-square or Fisher’s exact test for categorical variables as appropriate.

^β^Excluding the below adverse events. *Bonferroni adjusted *p*-values.

#### Thirty-day outcome (death and 30-day cumulative adverse events)

Despite the higher risk profile and in-hospital complication rates within the 24–48 h group, the 30-day outcomes did not differ significantly when compared to the 24 h group (11.7 vs. 11.3%, *p* = 0.9) (Table 2). Young males with bicuspid valves who had minimal use of contrast volume and no procedural-related complications were more likely to be discharged within 24 h than 24–48 h (Table 3). The >48 h had a significantly higher rate of 30-day death (2.1 vs. 0.1%, *p* < 0.001) and cumulative adverse events when compared to the 24 h (15.4 vs. 11.3%, *p* = 0.03). Non-bicuspid patients with low BMI, high surgical risk (STS ≥ 8), a low KCCQ score (<50), low LVEF, and severe mitral regurgitation that had procedure-related complications, were more likely to be discharged more than 48 h after the procedure (Table 4).

**TABLE 3:** Univariate and multivariable analyses of discharge within 24 h (vs. 24–48 h).

Variable	N	Discharge within 24 h vs. 24–48 h Univariate		Discharge within 24 h vs. 24–48 h Multivariable	
		Odds ratio (95% CI)	*P*-value	Odds ratio (95% CI)	*P*-value
Age (years)	1,529	0.97 (0.96–0.98)	<0.001	0.98 (0.97–0.99)	<0.001
Male sex	974	1.29 (1.04–1.59)	0.02	1.22 (0.98–1.52)	0.07
Body mass index	1,529	1.00 (0.98–1.02)	0.88	ψ	
STS score ≥ 8	129	1.08 (0.75–1.55)	0.69	ψ	
KCCQ score < 50	593	0.82 (0.67–1.02)	0.07	ψ	
LVEF < 30%	54	1.28 (0.74–2.23)	0.38	ψ	
Severe mitral regurgitation	32	0.90 (0.45–1.80)	0.77	ψ	
Severe tricuspid regurgitation	43	0.92 (0.50–1.68)	0.77	ψ	
Urgent procedure	191	0.79 (0.58–1.07)	0.12	ψ	
Bicuspid aortic valve	160	3.98 (2.65–5.92)	<0.001	3.60 (2.38–5.42)	<0.001
Use of intra-aortic balloon pump	11	2.34 (0.62–8.84)	0.21	ψ	
Contrast volume (ml) used	1,527	0.97 (0.94–1.00)	0.02	0.97 (0.94–1.00)	0.02
ICU length-of-stay	1,529	0.99 (0.95–1.02)	0.49	ψ	
Procedure-related complications	48	0.28 (0.14–0.54)	<0.001	0.33 (0.16–0.65)	0.001
Procedure-related life-threatening complications ^ψ^	8	0.12 (0.02–1.01)	0.05	ψ	

STS, society of thoracic surgeon; KCCQ, Kansas city cardiomyopathy questionnaire; LVEF, left ventricular ejection fraction. 1,527 observations were used in the multivariable analysis. ^ψ^ Dropped out of the analysis.

**TABLE 4:** Univariate and multivariable analyses of discharge within 24 h (vs. >48 h).

Variable	N	Discharge within 24 h vs. >48 h Univariate		Discharge within 24 h vs. >48 h Multivariable	
		Odds ratio (95% CI)	*P*-value	Odds ratio (95% CI)	*P*-value
Age (years)	1,709	0.98 (0.97–0.99)	<0.001	ψ	
Male sex	1,072	1.35 (1.11–1.65)	0.003	ψ	
Body mass index	1,709	1.03 (1.01–1.04)	0.003	1.04 (1.02–1.06)	
STS score ≥ 8	359	0.20 (0.15–0.27)	<0.001	0.31 (0.23–0.42)	<0.001
KCCQ score < 50	709	0.50 (0.41–0.61)	<0.001	0.59 (0.47–0.74)	<0.001
LVEF < 30%	150	0.27 (0.18–0.40)	<0.001	0.59 (0.37–0.96)	0.03
Severe mitral regurgitation	92	0.22 (0.12–0.37)	<0.001	0.55 (0.30–1.00)	0.05
Severe tricuspid regurgitation	100	0.29 (0.18–0.47)	<0.001	ψ	
Urgent procedure	232	0.68 (0.52–0.91)	0.01	ψ	
Bicuspid aortic valve	177	3.20 (2.27–4.52)	<0.001	2.18 (1.48–3.22)	<0.001
Use of intra-aortic balloon pump	11	2.93 (0.78–11.08)	0.11	ψ	
Contrast volume (ml) used	1,705	0.93 (0.91–0.95)	<0.001	ψ	
ICU length-of-stay	1,709	0.98 (0.95–1.02)	0.26	ψ	
Procedure-related complications	157	0.26 (0.17–0.38)	<0.001	0.25 (0.17–0.39)	<0.001
Procedure-related life-threatening complications ^ψ^	50	0.33 (0.17–0.63)	<0.001	ψ	

STS, society of thoracic surgeon; KCCQ, Kansas city cardiomyopathy questionnaire; LVEF, left ventricular ejection fraction. 1,553 observations were used in the multivariable analysis. ^ψ^ Dropped out of the analysis.

#### Predictors for outcome

**FIGURE 3:**
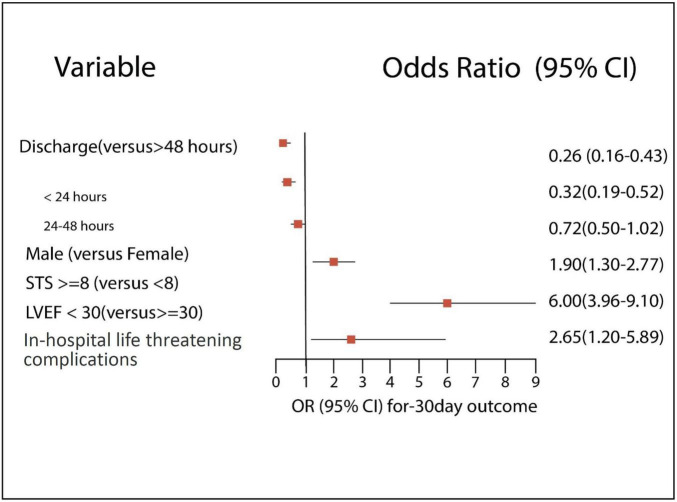
Significant predictors for 30-day outcomes.

Our study shows that independent poor prognostic factors of 30-day outcomes were discharge time (OR 0.26, 95% CI 0.16–0.43, *E*-value = 7.15, *P* = < 0.001 for discharge within 24 h), high surgical risk ≥8 (OR 1.90, 95% CI 1.30–2.77, *E*-value = 3.21, *P* < 0.001), low LVEF < 30 (OR 6.00, 95% CI 3.96–9.10, *E*-value = 11.48, *P* < 0.001), and procedure-related life-threatening complications (OR 2.65, 95% CI 1.20–5.89, *E*-value = 4.74, *P* = 0.04). Although the associations were not statistically significant, the female gender shows an association with a 30-day outcome (OR 0.72, 95% CI 0.50–1.02, *P* = 0.06) (Table 5 and Figure 3). A probability formula with a 30-day outcome using the significant predictors demonstrated a good ability to discriminate between those with and without a 30-day outcome (apparent area under the curve: 0.80; 95% CI: 0.77–0.83) (Figure 3). The internal validation by the bootstrapping method on entire cohort study showed an AUC (optimism-corrected AUC) of 0.78 (95% CI 0.75–0.81), which is slightly lower than the apparent AUC as expected and is well calibrated (optimism-corrected calibration-in-the-large of −0.05; the slope of 0.97) (Table 6).

**TABLE 5:** Univariate and multivariable analyses of 30-day outcome.

Variable	N	30-day outcome Univariate		30-day outcome Multivariable	
		Odds ratio (95% CI)	*P*-value	Odds ratio (95% CI)	*P*-value
**Discharge**					
<24 h	816	0.17 (0.10–0.27)	<0.001	0.26 (0.16–0.43)	<0.001
24–48 h	713	0.20 (0.13–0.32)	<0.001	0.32 (0.19–0.52)	<0.001
>48 h	893	1 (Reference)		1 (Reference)	
Age (years)	2422	1.02 (1.00–1.04)	0.03	ψ	
Male sex	1504	0.74 (0.54–1.02)	0.06	0.72 (0.50–1.02)	0.06
Body mass index	2422	0.99 (0.96–1.02)	0.35	ψ	
STS score ≥ 8	417	4.49 (3.24–6.22)	<0.001	1.90 (1.30–2.77)	<0.001
KCCQ score < 50	1002	1.97 (1.41–2.75)	<0.001	ψ	
LVEF < 30%	172	9.39 (6.49–13.62)	<0.001	6.00 (3.96–9.10)	<0.001
Severe mitral regurgitation	108	3.37 (2.02–5.64)	<0.001	ψ	
Severe tricuspid regurgitation	121	3.76 (2.33–6.06)	<0.001	ψ	
Urgent procedure	331	2.22 (1.53–3.23)	<0.001	ψ	
Bicuspid aortic valve	209	0.45 (0.21–0.97)	0.04	ψ	
Use of intra-aortic balloon pump	14	2.29 (0.51–10.34)	0.28	ψ	
Contrast volume (ml) used	2,418	1.07 (1.04–1.10)	<0.001	ψ	
ICU length-of-stay	2,442	1.02 (0.96–1.07)	0.53	ψ	
Procedure-related complications	168	2.48 (1.56–3.96)	0.001	ψ	
Procedure-related life-threatening complications ^ψ^	44	3.64 (1.72–7.70)	<0.001	2.65 (1.20–5.89)	0.04

STS, society of thoracic surgeon; KCCQ, Kansas city cardiomyopathy questionnaire; LVEF, left ventricular ejection fraction. 2,447 observations were used in the multivariable analysis. ^ψ^ Dropped out of the analysis.

**TABLE 6:** Performance of the formulated predictors of 30-day outcome.

Discrimination		Calibration	
Apartment AUC (95 CI%)	Optimism-corrected AUC (95% CI) *	Optimism-corrected Calibration-in-the-large *	Optimism-corrected Calibration slope *
0.80 (0.77, 0.87)	0.80 (0.76, 0.84)	−0.06	0.97

*Internal validation by the bootstrap method.

#### Inverse probability treatment weighting (IPTW)

**FIGURE 4:**
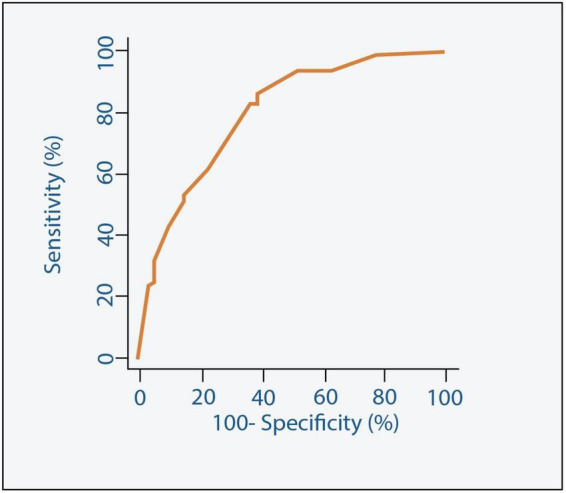
Receiver Operating Characteristic (ROC) curve along with the Area Under the ROC curve (AUC) for the model predicting 30-day outcome.

We balanced age, sex, STS score, KCCQ performance score and LVEF in a full inverse probability treatment weighting (IPTW) using propensity matching (Figure 4). The matched cohort included 1,428 patients, 714 patients discharged within 24 h, and 714 after 24 h. A total of 1,017 patients were unmatched or discarded from the analysis. We excluded procedural complications from the logistics regression analysis to address the possibility of selection bias for early discharge and to adhere as much as possible to a prospective methodology reflecting real-life.

In a matched population, an STS score of ≥8, LVEF of <30%, and KCCQ of >50 remain statistically significant poor prognostic predictors for 30-day cumulative outcomes of adverse events and death regardless of the discharge time (OR 2.18, OR 8.80, OR 1.74, *p* = 0.012, *p* < 0.001, *p* = 0.02, respectively). While age was not correlated with 30-day outcomes, females showed a non-significant strong association (OR 1.15, 95% CI 0.69–1.92, *P* = 0.05) (Table 7).

**TABLE 7:** Multivariable logistic regression of 30-day cumulative outcome after IPTW adjustment for patients discharge within 24 h and more than 24 h.

Variable	N	30-day Cumulative Outcome of 24 h vs. >24 h Multivariable analysis	
		Odds Ratio (95% CI)	*P*-value
Age (years)	1,428	1.08 (0.67–1.75)	0.750
Female sex	513	1.157 (0.69–1.92)	0.054
STS score ≥ 8	134	2.18 (1.19–4.01)	0.012
KCCQ score < 50	522	1.74 (1.07–2.85)	0.026
LVEF < 30%	58	8.80 (4.67–16.61)	<0.0001
Discharge group (24 h vs. >24 h)	1,428	0.97 (0.84–1.14)	0.132

STS, society of thoracic surgeon; KCCQ, Kansas city cardiomyopathy questionnaire; LVEF, left ventricular ejection fraction. 1,428 observations were used in the multivariable analysis. ^ψ^ Hosmer and Lemeshow test *p*-value = 0.908.

## Discussion

Our study is the largest scale study known to address the safety and the feasibility of early discharge for TAVR patients under general anesthesia. We compared the outcomes of 837 patients discharged within 24 h of admission to 713 patients discharged within 24–48 h to 897 patients discharged after 48 h. The day of discharge is intuitive and involves numerous factors, both subjective, such as patient’s frailty and social support, and objective. Our study offers a validated tool for patient selection in borderline cases and illustrates that select patients can be discharged within 24 h of GA-TAVR without increasing the risk of adverse events.

**FIGURE 5:**
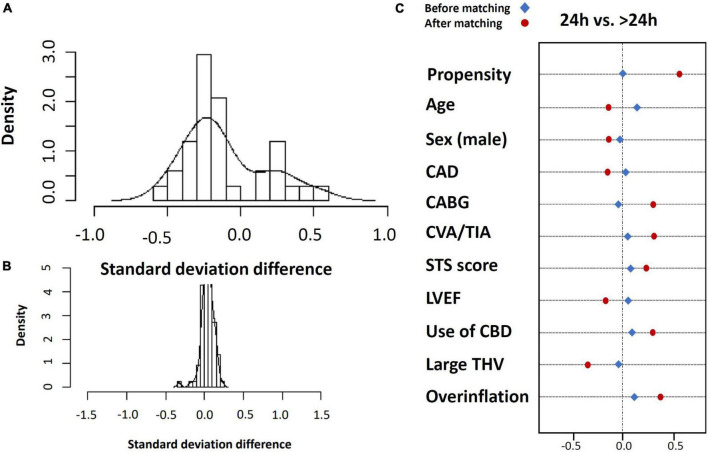
Standard deviation before **(A)** and after **(B)** inverse propensity treatment weighting (IPTW) using propensity matching and covariate balance for entire matched population **(C)**.

The length of hospitalization following TAVR has dramatically evolved over the years (Figure 5) primarily due to increased operator ability as well as technological improvements of valves and delivery systems (7). Efficient hospital turnover is critical in today’s healthcare environment, especially in light of the healthcare burdens that have risen from the COVID-19 pandemic (24, 25).

**FIGURE 6:**
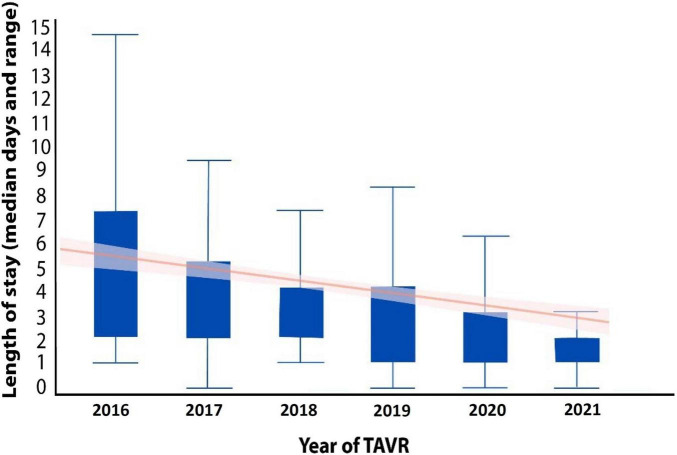
The median length of hospitalization of TAVR patients from 2016 to 2020.

## General anesthesia vs. conscious sedation

The majority of TAVR procedures were initially performed using general anesthesia to aid in intraprocedural TEE for accurate assessment of annular dimensions, valve deployment, and paravalvular leak (PVL), minimizing patient’s movement and immediate response following life-threatening complications such as cardiac tamponade, annulus rupture, aortic dissection, and valve migrations (26–28). Recent data from TAVR registries indicate that CS shares a similar outcome of all-cause mortality, stroke, myocardial infarction, infection, and acute kidney injury at 30 days with an advantage of shorter procedure time, shorter hospital stay, and lower costs (29, 30). Despite the ongoing trend, the use of GA is still performed in 33–63% of all TAVRs, with a wide variation across North America and Europe (31). In a French registry study from 2011, GA was used in 41% of patients, and in a German registry, the use of CS was reported to vary from 26 to 68% of patients, depending on the center’s experience (32–34).

Our medical center uses general anesthesia and TEE as routine practice for all TAVR patients. TAVR is primarily performed in a hybrid operating room with a dedicated team of anesthesiologists and echocardiologists. The patients are intubated close to the time of the procedure and sedated using short-acting medication. After an uncomplicated TAVR, the patients are transferred directly to the post-anesthesia cardiac unit (PACU) for early extubating and 4–6 h of complete bed rest followed by mobilization 2–4 h later. Fully recovered and functioning patients with non-major complicated TAVR and no postprocedural adverse events such as atrioventricular block, atrial arrhythmias, hemodynamic instability, or chest pain, stay for overnight observation and are discharged early the following day after they undergo routine post-operative testing which includes transthoracic echocardiography study, resting ECG, renal function tests and complete blood count. The patients are monitored during their entire stay in the hospital.

### Thirty-day outcome predictors and general anesthesia

The use of general anesthesia may be associated with a higher rate of adverse events and mortality in patients with comorbidities such as sleep apnea, chronic lung disease, illicit drug abuse, the use of supplementary home oxygen, and low cardiac ejection fraction (35, 36), which is incorporated in the STS score. To overcome it, we analyzed each component separately and found no interaction with the use of general anesthesia. Our study’s incidence of procedural-related life-threatening complications was not higher than reported in conscious sedation cohorts. We believe that these complications will probably result in longer LOH regardless of general anesthesia (36).

### Same-day discharge

There is growing evidence that selected low-risk TAVR patients can be safely discharged as early as the same day (37–41). In a recent publication by Krishnaswamy et al., regarding the feasibility and safety of same-day discharge, the time of initiating the TAVR had the highest odds ratio for the outcome (40). GA-TAVR patients require a careful pre-anesthesia evaluation and a relative prolonged postprocedural care than CS patients (41).

### Study limitation

The study is retrospective and based on data collections of patients’ electronic records and the 30-day outcome measures. The decision on exact discharge time may be affected by subjective factors such as social support and the patients frailty, which could not be thoroughly assessed using our study methodology. However, this was addressed by accounting for objective variables such as baseline comorbidities, related procedural characteristics, lab results, and the KCCQ, which is a performance scale model that, which integrates subjective assessment and subjective Variables as the Katz index of independence in activities of daily living which was validated in TAVR patients (42, 43). Moreover, we used a large sample size, three different comparison groups, and a multivariable strong statistical analysis and matching methods to avoid major bias in patient selection.

TAVR has changed dramatically over the past decade. With multiple commercially available valves and improvement in the delivery systems profiles, which have reduced procedural length and complications rates, patient safety has been enhanced (44–48). Our study included patients who underwent TAVR with new generation valves and delivery systems to reflect current day practices. However, our medical center utilizes balloon-expandable valves at a higher rate than self-expanding devices, which may decrease the external validity of our study (44, 45).

Length of stay (LOH) was affected during the late 2019 COVID outbreak and therefore necessitated rapid turnover and early discharge. This may impact our results, as other studies have previously reported (24, 25). We addressed it by including data from patients who underwent TAVR before and after the pandemic, as well as by including, and an intermediate discharge group of 24–48 h. This help to avoiding overlapping results. Furthermore, we demonstrate that the trend of minimizing LOS was initiated much before the pandemic, as we demonstrate.

We used propensity-score matching to compare patient population discharged at time points< and >24 h. It should be noted, however, that there were differences, particularly with the cohort discharged >48 h, that may have affected of our data. This included the baseline characteristics and higher rate of peri-procedural complications during the index hospitalization of those discharged at later time points.

Nevertheless procedural complications was not included in the logistics regression analysis to address the possibility of selection bias for early discharge and to adhere as much as possible to a prospective methodology reflecting real-life.

Our medical records are exposed to patients hospitalized only at Cedar’s networks and may therefore underestimate the true rehospitalization rate. However, the vast majority of patients (98%) attended the 30-day clinical visit and were interviewed by dedicated physicians and nurses to minimize the risk of missing information.

## Conclusion

Our study shows that independent poor predictors for a 30-day outcome with correlation to discharge time were high surgical risk, low KCCQ score, low LVEF, and life-threatening in-hospital complications.

Our formulated predictors were tested and validated using the entire study population and they demonstrated an ability to identify patients at risk of 30-day adverse events.

The trend of an optimized length of stay should cover all treatment protocols using general anesthesia and balance the need for a proper and safe post-procedural care such as a complete echocardiography study, a blood test for renal function, an ECG, and a clinical follow-up following re-mobilization.

## Data availability statement

The raw data supporting the conclusions of this article will be made available by the authors, without undue reservation.

## Author contributions

OK, VP, HJ, and RM conceptual the hypothesis, validated the results, and supervised all processes of the manuscript. EN, RN, TN, AS, SN, SK, ZA, AB, and AD assist in data collection, statistical analysis, and the final writing. MN and WC provided a detailed review and helped in the last draft. All authors contributed to the article and approved the submitted version.

## Abbreviations

AS: Aortic Stenosis
CS: conscious sedation
GA: general anesthesia
LVEF: Left ventricle ejection fraction
KCCQ: Kansas city cardiomyopathy questionnaire
LOS: Length of stay
PVL: paravalvular leakage
STS: Society of thoracic surgeon
TAVR: Transcatheter aortic valve replacement
TEE: Transesophageal echocardiography
THV: Transcatheter aortic valve.
